# Gender Differences and the Relationship of Motor, Cognitive and Academic Achievement in Omani Primary School-Aged Children

**DOI:** 10.3389/fpsyg.2018.02477

**Published:** 2018-12-17

**Authors:** Kashef Zayed, Petra Jansen

**Affiliations:** ^1^Department of Physical Education, Sultan Qaboos University, Muscat, Oman; ^2^Faculty of Psychology, Pedagogic and Sport Science, University of Regensburg, Regensburg, Germany

**Keywords:** working memory, mental rotation, motor performance, children, Oman

## Abstract

Until now, there has been no integrated study of the cognitive, motor and academic developments in children in the Arab world. In this study we investigated gender differences in those three areas in primary school-aged children in Oman and as well as the inter-relations between those three aspects of development. Ninety-five third graders completed four working memory tests, a mental rotation test and a motor test. Furthermore, the marks in math, science and Arabic language were registered. The result showed that there were small gender differences: Girls performed better in one of the working memory tests and boys in the coordination motor test. The study also showed that there were significant correlations between cognitive variables and academic performance, as well as two significant correlations between motor performance and marks in math and science. Marks in math correlated with the performances in the 20 m run and the coordination test, whereas the marks in science correlated with the ball-leg-wall test, the coordination test, and the endurance test. Regression analysis showed that all marks were predicted by the working memory and mental rotation performance as well as the motor ability. This means that academic achievement in Oman could be predicted by basic cognitive as well as motor abilities.

## Introduction

The main goal of this study was to investigate the development of primary school-aged children in Oman regarding their cognitive and motor development, as well as the difference between girls and boys in this respect. As far as we know, there have been no such studies on this topic in the Arabic Middle-East area. Those studies are necessary to understand the development of the children in non-western or Asian parts of the world, where almost all studies in this research area were conducted.

### Relation Between Cognitive and Motor Development and Academic Achievement

It is widely accepted that a lifelong positive relation exists between motor and cognitive performance (for example for the elderly, Colcombe and Kramer, 2003). Athletes perform better in executive functions (Vestberg et al., 2012) and show higher processing speed (Voss et al., 2010) and better attention performance (Hüttermann and Memmert, 2014) compared to non-athletes. A recent large-scale reanalysis of childhood fitness and inhibitory control (Raine et al., 2018) which used the records of 702 American boys and girls aged 8–11 strongly suggested that general physical fitness was associated with better discrimination accuracy as well as faster information uptake. The authors also concluded that aerobic fitness may benefit cognitive behavior and brain development.

Next in importance is the relationship between visual-spatial ability and the development of physical skills (motor development). Athletes have been shown to perform better in spatial tasks compared to physically inactive people (Pietsch and Jansen, 2012; Voyer and Jansen, 2017). More specifically, there appears to be a relationship between motor abilities and the ability to mentally manipulating objects in space (Shepard and Metzler, 1971) among pre-school children (Jansen and Heil, 2010) as well as adolescents (Jansen et al., 2018a). Furthermore, in two interventions studies (Blüchel et al., 2013; Pietsch et al., 2017) it was shown that in primary school-age children’s mental rotation performance could be improved with different motor-coordination trainings compared to a control group which received normal theoretical class room lessons or received a different motor activation program.

Regarding the effect of motor abilities or physical activity on school performance Castelli et al. (2014) summarized 20 relevant studies. They suggested that children with higher physical fitness were likely to be better academic performers as well. They were also less likely to miss classes. The authors concluded that “being physically active, having a lower BMI, and being physically fit were contributory to learning in the school environment” (Castelli et al., 2014, 139).

Summarized, both executive functions and visual spatial ability are related to motor development or physical activity. This is an important point because cognitive abilities are related to school success. Executive functions are relevant for performance for academic achievement (Röthlisberger et al., 2013) and ability is crucial for achievement in many school subjects such as natural sciences and mathematics (Nuttall et al., 2005; Lubinski, 2010; Newcombe et al., 2015).

### Gender Differences in Cognitive and Motor Development and Academic Achievement

The effects of gender on cognitive aspects investigated in this study–working memory and mental rotation ability–have been well studied in the western world. For example, regarding visual-spatial working memory a small but significant male advantage has been shown (Voyer et al., 2017). Only in the task of memory location a female advantage has been indicated. Age may be a relevant predictor, because gender differences appear first in adolescence. For the Corsi block-tapping test the estimated *d* was 0.17, indicating a small effect size for gender. Neuroscientific studies provide evidence that different neural networks are recruited by males and females while they solve different working memory tasks (Hill et al., 2014). In mental rotation abilities, gender differences favoring males is widely assumed (Voyer et al., 1995), an often cited assumption which was questioned by Jansen-Osmann and Heil (2007). In one study using a paper-pencil mental rotation test, fourth graders showed a small significant gender difference favoring boys independent of the stimuli material (animal drawings, letters, and cube figures), there was no effect in second graders (Neuburger et al., 2011). This is in line with a study of Titze et al. (2010) who showed widening gender difference in mental rotation performance between ages 9 and 10 years.

Concerning motor abilities, a recently published study showed differences between boys and girls aged 5–11 years (Roth et al., 2018): Girls were better than boys in balancing backward, stand-and-reach, and jumping sideways, whereas boys were faster than girls in running events. Several meta-analytical studies have examined possible gender differences in academically important factors such as mathematical ability, verbal skills, and spatial ability. Mathematical performance tended to be similar in boys and girls. Most of these results were obtained from performance tests or national tests.

Among the larger of such meta-analytic studies was conducted by Voyer and Voyer (2014) who focused on school- and college-marks, from which they isolated 502 effects sizes from 369 samples. Their results indicated a small but significant overall advantage for females, not only in language (where females are expected to outperform), but also in mathematics (which, along with science, is popularly seen to be a male bastion). The authors have suggested *inter alia* that the cultural stereotype of science and math as better suited for boys might create lower expectancy-value in the minds of girls, causing them to make less effort in science and math. It was also found from this study that for language and math, gender differences grew wider in middle and high school than it was in elementary school. The other relevant moderators were national origin of the study, racial composition as well as gender composition. The type of school, the type of grading and the year of the publication of the study were not relevant. Multiple explanations, social and biological ones, for the female advantage could explain the better school performance of girls over the last century. However, until now there is no research on how gender differences affect academic performance in Arab countries such as the Oman.

### Gender Differences and Cognitive Development in Oman

According to the Global Gender Gap Report, Oman is ranked 133 out of 144 countries with a score of 0.612 (0 represents total imparity and 1 total parity). The gender ranking was based on multiple factors such as women’s economic participation and opportunity, educational attainment, health and survival, and political empowerment. Factors that pushed down Oman’s gender parity ranking included women’s lower economic participation and opportunities, as well as their lower political empowerment. This occurred despite near parity in important parameters such as education, health, and survival levels.

Until now, the cognitive and motor development of children in Oman has not been investigated at all. There are only a few studies which are related to this research point, one of which found out that low birth weight in Omani children is a predictor of cognitive impairment at school-age. There were 17% of low birth weight and 12% of children between 7 and 11 years performed under low average performance for selected school marks (Islam, 2015). ADHD was another problem that affected Omani children’s school performance. It is the most discovered disorder among Omani pre-school children with 70.8% of all diagnosis (Al-Sharbati et al., 2016). The overall school-dropout rate for Omani children diagnosed with ADHD is 16.6% (Mirza et al., 2018). Education is free in Oman but attendance in school is not mandatory at any age.

Another study, which is related to the cognitive development and the topic of gender difference in children in Oman, was a study in which the performance of students was compared with a cohort of German students. German students outperformed Omani students in a test of cognitive processing speed and executive functions as well as in mental rotation. In both samples there was a gender difference in mental rotation performance favoring males compared to females, there was no gender difference in the cognitive performance test. In this study there was no correlation between the amount of physical activities and the mental rotation performance (Jansen et al., 2016), contradicting the hypotheses that physical activity is related to spatial cognition performance in some way (Voyer and Jansen, 2017).

## Hypotheses of the Study

### Gender Differences in Cognitive, Motor and Academic Achievement

According to the results in studies with western samples, gender difference favoring boys might be expected in visual-spatial working memory task as in the mental rotation task, but the onset of possible gender differences in both areas is not clear yet. Beside this, girls should show better academic performance and possible gender difference in motor task should depend on the kind of task.

### Relation Between Cognitive, Motor Development and Academic Achievement

It is hypothesized that motor performance is related to working memory and mental rotation performance in some way, and is also related to academic achievements, specifically in mathematics, science and language.

## Methods

### Participants

The participants were 95 children (boys: 48, mean age: *M* = 8.00, *SD* = 0.41, girls: 47, mean age: *M* = 7.95, *SD* = 0.55) from the third grade of a public school in Muscat, Oman. Boys (*M* = 14.91, *SD* = 2.54) and girls (*M* = 14.98, *SD* = 2.80) did not differ in their BMI, *t*(118) = –0.118, *p* = 0.907. All children were Omani citizens. All parents gave written informed consent and the data were processed anonymously. The experiment was conducted according of the guidelines of the declaration of Helsinki. The study was ethically approved through the Deanship of Research at Sultan Qaboos University (IG/EDU/PHED/17/02).

### Materials

For the motor test, the General Motor Test for children aged 6–11 years was used; for the mental rotation test, the MRT-K-Animal Version; and for the working memory test the Digit span forward and backward test as well as the Corsi block-tapping test were used.

#### Motor Test

The General Motor test includes testing of speed, two precision tasks, springiness, endurance, and a coordination task under time pressure. For measuring *speed*, the children had to complete two 20 m runs, and the better performance of the two was used. *Precision* was measured with (a) target-throw measurement and (b) ball-leg-wall throw. For (a), the subject threw a tennis-ball from 3 m distance to a specific marked area on the wall. According to the precision of hitting three different quadrants in two series of five throws, the child received 0–3 points. Concerning the second task (b), children turned with the back to the wall in a three-meter distance and threw a gymnastic ball through their legs onto the wall. According to a classification scheme the children received between zero and five points; the sum of points for ten throws was registered. *Springiness* was measured by the task to push a 1 kilogram weighted medicine ball as far as possible. Endurance was measured with the performance in a 6 min run. The *whole body coordination* was tested using castle boomerang test (Jansen et al., 2018b). Various motor tasks (somersault, jumping over castles, etc.) had to be completed under time pressure. All points were transformed to percent-ranges and z-values. For the overall motor score the six z-values were summed up and divided by six.

#### Mental Rotation Test

**FIGURE 1:**
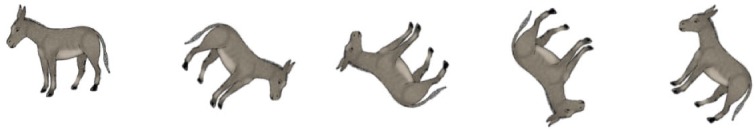
Example of one item of the MRT-K-Animal Version.

The child-adapted mental-rotation-test (MRT-K-Animal Version) consists of 16 tasks in general. Four tasks were presented per DIN-A4-sized, landscape-formatted sheet of paper. Each task included five items, pictures of animal drawings, one standard item on the left side and four comparison items on the right side. Two out of the four comparison items were non-mirror images but picture-plane-rotated versions of the target, which were called “correct” items. The other two items were mirror images picture-plane rotated versions of the target; which were called “incorrect” items. The items were rotated in the following angular disparities: 45°, 90°, 135°, 225°, 270°, and 315°. (see Figure 1). Children were required to mark the correct two target pictures. One point was given, when both correct items were marked.

Because Omani children are not used to mental rotation tests, time limit restriction was removed, despite which their performance was poor. Therefore, to train the children on the concept of mental rotation, two training items were presented and explained to the children. The test was repeated two months after the first test but in the standard version with a time limit of four minutes for the solution of the 16 items.

#### Working Memory Tests

Corsi block-tapping test forward and backward was used. The Corsi block-tapping test forward measures the visuo-spatial sketchpad; the Corsi block-tapping test backward component tests the central executive of working memory. The test hardware consists of wooden blocks fixed on a wooden panel. The examiner shows the child a sequence of blocks. This sequence, which begins with the repetition of two numbers, must be repeated either in the forward or backward direction by tapping it. The test is finished when a child is unable to repeat the sequence three times. The length of the block sequences that could be repeated twice is the maximum score of each participant. The reliability of this test is between 0.81 and 0.89.

Digit span forward and backward test was also used. This is part of the Hamburger-Wechsler-intelligence-test for children (Petermann and Petermann, 2010). Digit span forward test measures the phonological loop; Digit span backward test measures the verbal working memory processes. It is the task of the children to repeat the numbers, which varied in length, that are verbally given by the examiner in forward or backward direction. The task is finished when a sequence is not repeated successfully from the child. Maximum score for the forward task is 32 with a split-half reliability of 0.76; the maximum score for the test backward is 16 with a split-half reliability of 0.78.

### Procedure

First of all, relevant demographic data were retrieved and the participants’ weight and height measured. After this, the working memory tests were conducted, first the Digit span forward and backward test and after this the Corsi block-tapping test. These tests were completed in singles sessions. After this the mental rotation test was applied in group-sessions from 10 to 12 kids. On another day in the week the motor test was conducted with the children. The academic marks in Arabic, Mathematics and Science were given by the teachers through the school Principal. The teacher assess the learning of the children in several ways including written and oral exams.

Time taken: The working memory tests lasted approximately 45 min (excluding the mental rotation test which had no time limit). The motor tests lasted about 30 min.

### Statistical Analysis

Concerning the effect of gender on cognitive performance and motor abilities one univariate analysis of variance was conducted with the dependent variable “points in the mental rotation test” and three multivariate analyses, one for the working memory measurements, one for the motor ability tests and one for the marks with the factor gender were executed.

Furthermore, a correlation analysis was conducted with the relevant cognitive (four working memory task, on mental rotation task), marks (math, science, and Arabic) and the overall motor value. Significance level was corrected according to the Bonferroni-method and *p* was set to 0.00625. The correlation was qualified by another correlation analysis between math and science marks and the different motor tests. Significance level was corrected according to the Bonferroni-method and *p* was set to 0.00714.

## Results

### Gender Differences

#### Cognitive Measurements

The univariate analysis for the mental rotation performance showed no main effect of “gender”, *F*(1,93) = 0.986, *p* = 0.323, partial η^2^ = 0.010. Concerning the working memory test, the result of the MANOVA showed an overall effect of gender, *F*(4,90) = 3.203, *p* = 0.017, partial η^2^ = 0.125, but gender seemed to have only a significant effect for the Digit span forward test, *F*(1,93) = 11.167, *p* = 0.001, partial η^2^ = 0.107. Girls (*M* = 7.12, *SD* = 0.96) showed a better performance than boys (*M* = 6.42, *SD* = 1.21).

#### Motor Abilities

The result of the MANOVA showed no overall effect of gender, *F*(6,88) = 1.653, *p* = 0.142, partial η^2^ = 0.101. All z-values were between 90 and 107. The further analysis only indicated a significant better performance in the castle-boomerang test for boys (*M* = 99.708, *SD* = 9.67) compared to girls (*M* = 94.89, *SD* = 10.14), *F*(1,93) = 5.605, *p* = 0.02, partial η^2^ = 0.057.

#### Marks

The result of the MANOVA showed no overall effect of gender, *F*(3,91) = 1.759, *p* = 0.161, partial η^2^ = 0.055 and no effect in none of the single marks (all *p* > 0.05, all partial η^2^ < 0.033).

### Relation Between Mental Rotation, Working Memory, Motor Abilities and Grades in Science, Mathematics and Arabic

#### Correlation-Analyses

First of all, there was a high correlation between the performance in the first mental rotation test without time limit and the second one with time limit, (*r* = 0.466, *p* < 0.001). We included in all analyses (correlation and regressions) the first mental rotation without time limit because this test was conducted at the same time as the other tests. The repetition of the regressions with the mental rotation test without time limit indicated the almost same predictors as significant. However, mental rotation was not a significant predictor anymore for marks in Science and Arabic.

Table 1 shows the correlation between the variables mentioned above.

**Table 1:** Correlation between maths, science, and Arabic language marks, cognitive performance and motor abilities.

	Digit span forward test	Digit span backward test	Corsi block-tapping test forward	Corsi block-tapping test backward	Mark Arabic	Mark Maths	Mark Science	Mental Rotation	Motor Test
Digit span forward test	1	0.420^∗^	0.221	0.104	0.366^∗^	0.241	0.262	0.112	0.095
Digit span backward test		1	0.229	0.173	0.350^∗^	0.328^∗^	0.376^∗^	0.203	0.045
Corsi block-tapping test forward			1	0.302^∗^	0.311	0.267	0.304^∗^	0.254	0.009
Corsi block-tapping test backward				1	0.309^∗^	0.291^∗^	0.323^∗^	0.126	-0.086
Mark Arabic					1	0.757^∗^	0.740^∗^	0.285^∗^	0.261
Mark Maths						1	0.865^∗^	0.425^∗^	0.311^∗^
Mark Science							1	0.295^∗^	0.352^∗^
Mental Rotation								1	-012
Motor test									1


*^∗^p < = 0.004.*

The analyses revealed correlations between cognitive variables, such as the Digit span forward and backward test, and the Corsi block-tapping test forward and backward. Further, there were significant correlations between cognitive variables and academic achievement as well as two significant correlations between motor performance and marks in math and science. Because of these correlations, the relations of the grades to the specific motor tests were investigated further (see Table 2).

**Table 2:** Correlation between maths and science marks, and motor abilities.

	Mark Math	Mark Science	20 meter run	Goal Throw	Ball leg wall	Coord. test	Medicine Ball	6 min run
Mark Math	1	0.865^∗^	0.344^∗^	0.145	-0.129	0.278^∗^	0.102	0.281
Mark Science		1	0.344^∗^	0.110	-0.034	0.316^∗^	0.126	0.295^∗^
20 m run			1	0.245	0.127	0.443^∗^	0.174	0.091
Goal throw				1	0.057	0.083	0.150	-0.023
Ball-leg-wall					1	0.048	0.267	0.101
Coordination test						1	0.269	0.091
Medicine ball							1	0.011
6 min run								1


*^∗^p < = 0.004.*

Mark in math correlated significantly with the performances in the 20 m run and the coordination test, whereas the mark in science correlated with the 20 meters run, coordination test and endurance test.

#### Regression-Analyses

All regression analyses were calculated for the respective academic marks and the predictors: motor ability (overall score), Corsi block-tapping test forward and backward, Digit span forward and backward test, and first mental rotation performance without time limit.

##### Math marks

Multiple regressions were used to test if cognitive or the motor ability significantly influenced the math grade. The results of the regression indicated that three predictors predicted 39.6% of the variance (corrected *R^2^* = 0.355), *F*(6,88) = 9.616, *p* < 0.001). It was found that Corsi block-tapping test backward (ß = 0.222, *p* = 0.013), mental rotation (ß = 0.346, *p* < 0.001), and motor ability (ß = 0.320, *p* < 0.001), significantly predicted the grade in math.

##### Science marks

The multiple regression results show that 39.2% of the variance (corrected *R^2^* = 0.357) is explained by predictors Digit span backward test, (ß = 0.222, *p* = 0.018, Corsi block-tapping test backward (ß = 0.258, *p* = 0.004), and motor ability (ß = 0.398, *p* = 0.000), *F*(7,87) = 0.9383, *p* < 0.001).

##### Arabic marks

The multiple regression results show that 35.2% of the variance (corrected *R*^2^ = 0.307) is explained by predictors Corsi block-tapping test backward, (ß = 0.226, *p* = 0.015), and motor ability (ß = 0.255, *p* = 0.004), *F*(6,88) = 7.956, *p* < 0.001).

#### Regression-Analyses Due to Multicollinearity

Because of the high correlation between the Digit span forward and backward test performance as well as the performance in the Corsi block-tapping test forward and backward, multi-collinearity exists. For this, the three regression analyses were repeated with the predictors Corsi block-tapping test backward, Digit span backward test, mental rotation and motor ability.

##### Math mark

The results of the regression indicated that all four predictors predicted 38.9% of the variance (corrected *R^2^* = 0.361), *F*(4,90) = 14.299 *p* < 0.001). It was found that Corsi block-tapping test backward (ß = 0.240, *p* = 0.006), Digit span backward test (ß = 0.199, *p* = 0.022), (ß = 0.358, *p* < 0.001), and motor ability (ß = 0.327, *p* < 0.001), significantly predicted the grad in math.

##### Science mark

The multiple regression results show that 38.3% of the variance (corrected *R^2^* = 0.355) is explained by predictors Digit span backward test, (ß = 0.268, *p* = 0.002), Corsi block-tapping test backward (ß = 0.282, *p* = 0.001), mental rotation (ß = 0.209, *p* = 0.016), and motor ability (ß = 0.367, *p* < 0.001), *F*(4,90) = 13.93, *p* < 0.001).

##### Arabic mark

The multiple regression results show that 29.9% of the variance (corrected *R^2^* = 0.268) is explained by predictors Corsi block-tapping test backward, (ß = 0.263, *p* = 0.005), Digit span backward test, (ß = 0.251, *p* = 0.007), mental rotation (ß = 0.204, *p* = 0.026) and motor ability (ß = 0.275, *p* = 0.003), *F*(4,90) = 9.59, *p* < 0.001).

## Discussion

Our results could be summarized as follows: First, there were no relevant gender differences in cognitive, motor, and academic development in primary school-aged Omani children. There was only one significant effect in one working memory measurement that favored girls and one significant effect in the coordination test that favored boys. Second, in both genders, there was a detectable relationship between cognitive measurements and academic achievement, as well as between motor ability and performance in mathematics and science.

### Gender Differences in Cognitive, Motor and Academic Achievement in Omani Primary School-Aged Children

The missing gender differences in cognitive development in Omani children of the third grade are partly in line with the literature. For example, children in our study did not show gender differences in paper mental rotation test. However, they were around 8 years old, while gender differences in such a test are expected to become significantly detectable between the ages 9 and 10 years (Titze et al., 2010; Neuburger et al., 2011). Because gender differences in Omani students solving a mental rotation test could be detected (Jansen et al., 2016), the development of gender differences in this kind of spatial ability in Omani and Middle Eastern population might arise later somewhere between late primary school-age, and like in other populations, keep widening through adolescence and adulthood. Until now, no study has investigated this developmental course in Oman or its underlying mechanism. Underlying forces for such gender-based changes might be biological, triggered by hormonal changes during adolescence (Quaiser-Pohl et al., 2016) or socio-cultural, with the development of stereotype thinking (Newcombe et al., 1983). No formal research has explored the gender-based divergence in cognitive abilities during various growth phases of Omani children, or the impact of sociocultural factors in generating and shaping gender biases among them.

The participants in our study had low BMI. Low BMI is generally associated with late onset of puberty. Regarding the missing gender differences for example in visual-spatial memory, this is in line with the meta-analysis of Voyer et al. (2017), which marked the onset of gender differences favoring males in adolescence. The only gender difference in motor abilities among our subjects was in a coordination test whose outcome favored boys. This contradicts the study of Roth et al. (2018) mentioning gender difference favoring boys only in running tasks. In general, this comparison has to be considered with caution because different tests are used. The other missing gender differences in motor abilities might be because of the desert climate of Arabian Peninsula which remains hot and dry for eight months a year, which prevents Omani children from being very active outdoors.

In contradiction to the result, that girls are always better in school performance measured with marks, the result presented here did not show any difference. It is astonishing that despite mounting research evidence, such an assumption is still sustained among many educationists, policy makers, popular press, who in turn mold the convictions of parents and children. This bias is perhaps preventing many talented girls from taking up scientific careers. Voyer and Voyer (2014) showed ethnic composition to be a significant moderator for global measures of gender difference in academic achievement when non-US samples were included in the data. Because ethnic composition did not differ in our sample, boys might not have shown worse performance. Voyer and Voyer (2014) did also not find an influence of the kind of school, private vs. public, which suggests that the bias cuts across socioeconomic status as well. Our subjects were studying in a government-run school and were from families of lower-than-average socioeconomic status. This has to be taken into account while investigating in more detailed school achievement in the Arab World. Furthermore, the role of stereotype on promoting gender-gap has to be investigated in detail: the similarity of parents in Oman to western parents in their conviction that boys are able to learn math and science better than girls.

### Relation Between Cognitive, Motor Development and Academic Achievement in Omani Primary School-Aged Children

Our result shows that academic achievement could be predicted by basic cognitive mechanisms such as working memory and visual spatial abilities, as well as motor abilities. This holds true for the three academic subjects taught in Oman: mathematics, science, and language (Arabic). Thus, basic cognitive as well as motor variables are relevant for the school success in children in Oman. This new result is in line with the studies in Western societies, which also show positive relation between academic achievement and executive functions (Röthlisberger et al., 2013), mental rotation and grades in math (Blüchel et al., 2013) and physical fitness and academic achievement (Castelli et al., 2014). It must be considered, however, that we did not investigate physical fitness as a whole, only motor abilities (Haga, 2008). In addition our results show a correlation between the mark in science and the performance in the 6 min run, an endurance test, which can be considered as a test of physical fitness.

In contrast to the studies in western societies there was no correlation among our subjects’ between motor abilities and mental rotation performance. Being a fairly new result this needs critical scrutiny. Thus, initially we suspected a confounder to cause this unusual finding because the first mental rotation test had been applied in a non-speeded manner (with no time limit) and motor abilities require some speediness. Therefore, we reanalyzed our data and correlated the second speeded mental rotation test with different motor tests. As none of the correlations was significant, the confounder was excluded. The performance of our subjects (grade 3 pupils) in the second mental rotation test was exactly between the performance of German children in grade 2 and 4, who also had animal pictures as stimuli (Neuburger et al., 2011). This higher difficulty was carved out in an African primary school-aged sample. Children in Cameroon were not able to solve this test, which was not the case in the sample presented here. Possible reasons for the missing relation between motor abilities and mental rotation might be the minor physical activity of Omani children especially the minor engagement in activities which require a spatial or rotational enrollment (Jansen et al., 2018b). Due to the hot weather in Oman it is supposed that children living there have not many opportunities to play outside such games as soccer, which improves spatial cognition. Unfortunately relevant data relating to this assumption are missing. Additionally, the traditional view of gender education might especially girls prevent to engage in sports.

### Limitations and Future Research

The first goal of the study was to investigate gender difference in cognitive, motor and academic achievement in primary school-aged children, and the second goal to investigate the relation among those variables. The study gives a hint that gender differences in performance at the studied age are not common in most of the retrieved variables in Oman, an Arabian peninsular country. This finding goes against the picture of male-dominance that is accepted as the norm in the Eastern Mediterranean region. Second, our study showed that academic achievement in Omani children could be predicted by basic cognitive and motor data. This suggests that the training of these components may improve academic achievement and should be considered for inclusion in the educational system.

The study is limited by the fact that only basic cognitive and motor functions were investigated. Further studies should consider to widen this, and to integrate the investigation of the inhibition as well as cognitive flexibility development. Further, the developmental course has to be considered in more detail, investigating children and adolescents across all school-ages. In addition, the underlying mechanism of the relation between cognitive and motor performance on the one side, and academic achievement on the other, could be investigated in detail.

## Author Contributions

KZ helped in designing the study, organized the data acquisition, helped in analyzing the data, and discussed the first draft of the paper. PJ designed the study, analyzed the data, and wrote the first draft of the paper.

## Conflict of Interest Statement

The authors declare that the research was conducted in the absence of any commercial or financial relationships that could be construed as a potential conflict of interest.
